# Effectiveness and safety of carbon ion radiotherapy for stage III non-small cell lung cancer: a single-center retrospective study

**DOI:** 10.3389/fonc.2026.1861846

**Published:** 2026-06-30

**Authors:** Xiaoli Zhao, Bole Wang, Yihe Zhang, Pengqing Li, Xuexue Liang, Qinli Gong, Ling Tian, Tianyan Qin, Xuelian Chen, Zhenglin Li, Peng Nie, Yanshan Zhang, Yancheng Ye

**Affiliations:** 1 Department of Public Health, Gansu University of Chinese Medicine, Lanzhou, Gansu, China; 2 Department of Radiotherapy, Gansu Wuwei Tumor Hospital, Wuwei, Gansu, China; 3 Follow-up Office, Gansu Wuwei Tumor Hospital, Wuwei, Gansu, China

**Keywords:** adverse reactions, carbon ions, efficacy, non-small cell lung cancer, radiotherapy

## Abstract

**Objective:**

To evaluate the clinical efficacy and adverse reactions of carbon ion radiotherapy (CIRT) for stage III non-small cell lung cancer (NSCLC), and to analyze factors influencing patient prognosis.

**Methods:**

A retrospective analysis was conducted on the clinical data and follow-up records of 82 patients diagnosed with stage III NSCLC who underwent CIRT between March 2020 and March 2024. The total CIRT dose administered ranged from 48 to 78 Gy (relative biological effectiveness, RBE), with a median follow-up duration of 31.5 months. The Kaplan-Meier method was used to calculate overall survival (OS), progression-free survival (PFS), and local control (LRC) rates. The Cox regression model was employed to analyze prognostic factors, and Firth’s Cox model was utilized for subgroup analysis. Adverse events were assessed according to the Common Terminology Criteria for Adverse Events (CTCAE) version 5.0.

**Results:**

Among the 82 patients, the 1-year OS, PFS, and LRC rates were 85.4%, 79.8%, and 95.3%, respectively; the 2-year OS, PFS, and LRC rates were 65.9%, 50.8%, and 82.9%. The median PFS was 26 months. Grade 1 radiation pneumonitis occurred in 5 patients (6.1%); grade 1 radiation esophagitis was observed in 36 patients (43.9%)with 3 patients (3.7%) experiencing grade 2; grade 1 radiation dermatitis was noted in 2 cases (2.4%), grade 2 in 1 case (1.2%), and grade 3 in 1 case (1.2%). Grade 3 or higher adverse reactions were primarily hematologic, with grade 3 lymphopenia being the most common (29 cases, 35.37%). Multivariate analysis revealed that CIRT combined with immunotherapy was an independent favorable factor for improving OS (*P* = 0.025). The results of subgroup analysis also indicate that the survival benefits of combined treatment are highly consistent across different subgroups.

**Conclusion:**

The use of carbon ion therapy for stage III NSCLC can achieve significant survival benefits, and the adverse reactions are manageable. CIRT combined with immunotherapy is an independent factor for improving patient OS; however, further prospective studies are still needed to validate this association and determine the optimal treatment strategy.

## 1 Introduction

Currently, lung cancer remains the leading cause of cancer incidence and mortality worldwide, accounting for approximately 12.4% and 18.7% of all cancer cases and deaths (1). Non-small cell lung cancer (NSCLC) is the predominant histological type. Stage III NSCLC is highly heterogeneous, characterized by a high tumor burden and complex anatomical structures, with current treatment options being limited (2). For inoperable stage III NSCLC, chemotherapy combined with radiotherapy represents the standard treatment (3). In recent years, the introduction of immunotherapy and targeted therapy has improved clinical outcomes for some patients to a certain extent (4–6).

However, in conventional photon radiotherapy, dose escalation is constrained by the tolerance of surrounding normal tissues, which results in limited locoregional control (LRC) rates. Concurrent chemoradiotherapy can enhance radiosensitivity; however, its significant adverse effects often lead to treatment interruptions or dose reductions (7). Due to its unique Bragg peak characteristics, carbon-ion radiotherapy (CIRT) enables the precise delivery of high doses to the tumor target while significantly minimizing damage to surrounding normal tissues (8, 9). Its high relative biological effectiveness (RBE) effectively induces double-strand breaks in tumor cell DNA and possesses potent cytotoxicity even against radiation-resistant cells (10, 11). These characteristics provide CIRT with the theoretical potential to improve LRC while reducing adverse effects (12).

Since CIRT technology was first applied clinically in Japan in the 1990s, it has gradually gained acceptance worldwide. Although China entered this field relatively late, it has experienced rapid development in recent years (13). In 2019, the first domestically produced carbon-ion therapy system in China was approved for market release and subsequently put into clinical use in Wuwei, Gansu Province. Subsequently, systems were established and began operation at multiple centers, including Lanzhou, Hangzhou, and Putian. The clinical application of domestically produced carbon-ion systems in patients with stage III NSCLC is still in its early stages, characterized by a lack of long-term prognostic analyses based on the Chinese population and systematic evaluations of various comprehensive treatment regimens, whether combined with chemotherapy, immunotherapy, or radiotherapy alone.

Therefore, this study focuses on patients with stage III NSCLC who have undergone domestic CIRT to analyze their long-term prognosis and assess the differences in treatment efficacy across various therapeutic strategies. The aim is to provide evidence-based guidance for the optimized application of CIRT in the management of stage III NSCLC.

## 2 Material and methods

### 2.1 Study design and patients

This study is a retrospective clinical study that enrolled patients with stage III NSCLC who underwent CIRT treatment at Wuwei Cancer Hospital in Gansu Province between March 2020 and March 2024. Clinical staging was determined based on the TNM staging system outlined in the 8th edition of the American Joint Committee on Cancer (AJCC) Cancer Staging Manual (14). Inclusion criteria were: patients with histopathologically confirmed NSCLC; clinical stage III; and patients with inoperable tumors or who refused surgery. Exclusion criteria were: patients with concurrent primary tumors; patients with incomplete clinical data; and/or patients unwilling to cooperate with follow-up. Consequently, a total of 82 patients were enrolled. This study was approved by the institutional ethics committee, approval number: 2021-36.

### 2.2 Treatments

Depending on the specific location of the tumor, the patient is positioned in the supine or prone position and immobilized using a vacuum pad combined with a non-perforated thermoplastic film. If oblique-field irradiation is required, the patient is rotated along the vertical axis to the appropriate angle before the CT scan is performed. The scan range extends from the cricothyroid membrane to the lower margin of the L1 vertebra, with a slice thickness and interval of 3 mm. Respiratory-gated CT images are acquired at end-expiratory, and a four-dimensional CT (4D-CT) scan is performed simultaneously to quantify the amplitude of respiratory motion. The CT images are transferred to the ciPlan V2.0 system for target delineation.

The gross tumor volume (GTV) is defined as the gross tumor visible on imaging. The clinical target volume (CTV) includes the ipsilateral hilar and mediastinal prophylactic lymphatic drainage regions. The internal target volume (ITV) is defined as the range of CTV motion observed on 4D-CT. Planning target volume (PTV) 1 is the ITV expanded by 3–5 mm, with a prescribed dose of 48 Gy (RBE) delivered in 12–16 fractions; PTV2 is defined as a 5-mm margin around the GTV as delineated by 4D-CT, used for local dose escalation to the primary tumor, with a prescribed dose of 20–28 Gy (RBE) delivered in 4–7 fractions. The total dose for CIRT is 48–78 Gy (RBE), with a fraction dose of 2.38-6.60 Gy (RBE) per fraction.

During treatment, irradiation of PTV1 is performed first, followed by sequential irradiation of PTV2. The final dose delivered to the positive lymph nodes and the prophylactic lymphatic drainage area reaches 48 Gy (RBE), while the total dose to the primary tumor is between 68 and 76 Gy (RBE). The dose limits for critical organs adhere to the standards set by the National Institute of Radiological Sciences (NIRS) in Japan: Dmax for the main bronchus is < 60 Gy (RBE), Dmax for the esophagus is < 50 Gy (RBE), and Dmax for the spinal cord is < 30 Gy (RBE). In this study, the RBE was calculated using the Mixed Beam Linear-Quadratic Model (MBM-LQ). The model parameters were derived from HSG cell survival assays based on the linear-quadratic (LQ) model. The depth-dependent RBE profiles corresponding to 10% cell survival were subsequently obtained. These RBE depth profiles were then applied to the design of the range modulation device and to treatment plan optimization.

The decision to combine CIRT with systemic therapy is made by the medical team following a comprehensive assessment of the patient’s condition. Systemic therapies primarily include chemotherapy, targeted therapy, and immunotherapy. Common chemotherapy regimens comprise the TC regimen (taxane + carboplatin), the TP regimen (taxane + cisplatin), and the PP regimen (pemetrexed + platinum). Targeted therapy mainly involves EGFR-TKI drugs such as osimertinib and gefitinib, while immunotherapy typically includes PD-1 inhibitors such as tislelizumab, sintilimab, and pembrolizumab.

### 2.3 Follow-up

Follow-up data were primarily obtained through patient-initiated mail-in reports, telephone follow-ups, and records from in-person follow-up visits. Patients were followed up every 3 months during the first year after treatment completion, every 6 months during the second and third years, and annually thereafter. Follow-up evaluations included chest CT scans and blood tests, with brain MRI and PET-CT scans added as needed. The last follow-up date was March 2026, with a median follow-up duration of 31.5 months.

Clinical efficacy endpoints included overall survival (OS), progression-free survival (PFS), and LRC. OS was defined as the time from the start of radiotherapy to death from any cause or the last follow-up; PFS was defined as the time from the start of radiotherapy to disease progression or death from any cause; LRC is defined as the time from the start of radiotherapy to local or adjacent regional recurrence. Adverse events were graded from grade 1 to grade 5 according to Common Terminology Criteria for Adverse Events (CTCAE) version 5.0 (15), and acute adverse events were defined as those occurring within 3 months of the start of CIRT.

### 2.4 Statistical analysis

Survival analysis for OS, PFS, and LRC was conducted using the Kaplan-Meier method, with survival curves plotted accordingly. Univariate analysis was performed utilizing the log-rank test, which incorporated variables such as gender, age, AJCC stage, histological subtype, Karnofsky Performance Status (KPS) score, history of chemotherapy, targeted therapy, immunotherapy, and surgical intervention, in addition to CIRT dose and combination therapy (CIRT combined with chemotherapy, targeted therapy, or immunotherapy). This analysis aimed to identify prognostic factors influencing OS, PFS, and LRC rates. Variables with *P* ≤ 0.10 in the univariate analysis were subsequently included in a Cox proportional hazards regression model for multivariate analysis (16). The subgroup analysis of OS was conducted based on predefined baseline characteristics. Interaction terms were introduced into the Cox regression model of the overall cohort to evaluate the heterogeneity of treatment effects across different subgroups by assessing the interaction *P*-values. Subsequently, Firth’s Cox model was applied independently within each subgroup to calculate the hazard ratios (HR) and their 95% confidence intervals (CI) between treatment groups. The *P*< 0.05 indicates statistical significance. All data analyses were conducted using R version 4.5.2.

## 3 Results

### 3.1 Patient characteristics

This study included a total of 82 patients, with detailed patient characteristics presented in **Table 1**. The median follow-up duration was 31.5 months (range: 2–66 months), and the median age of participants was 65 years (range: 26–85 years). Among the cohort, there were 70 male patients (85.37%) and 12 female patients (14.63%). The median KPS score was 90 points (range: 40–90 points), with 38 patients (46.34%) having a history of smoking. The pathological subtypes identified were adenocarcinoma (31 cases, 37.80%), squamous cell carcinoma (44 cases, 53.66%), other types (3 cases, 3.66%), and unspecified subtypes (4 cases, 4.88%). Central lung cancer was identified in 53 cases (64.64%), peripheral lung cancer in 22 cases (26.83%), and interstitial pneumonia in 7 cases (8.54%). Clinical staging was classified as IIIA in 26 cases (31.71%), IIIB in 36 cases (43.90%), and IIIC in 20 cases (24.39%). Regarding genetic testing results, no data were available for 46 cases (56.1%); no significant mutations were detected in 10 cases (12.2%). Specific mutations included EGFR in 9 cases (10.98%; exon 19 deletion in 5, exon 21 mutation in 3, exon 18 mutation in 1), TP53 in 6 cases (7.32%), and KRAS in 4 cases (4.88%). PD-L1 TPS expression levels were <1% in 2 cases, 1%–49% in 3 cases, and ≥50% in 2 cases.

**Table 1:** Characteristics of 82 patients treated with carbon‐ion radiotherapy (CIRT).

Characteristics		Number	%
Age (years)	Median (range)	65(26~85)	−
	≥65	46	56.10
	<65	36	43.90
Gender	Male	70	85.37
	Female	12	14.63
KPS score	Median (range)	90(40~90)	−
Histology	Adenocarcino	31	37.80
	Squamous cell carcinoma	44	53.66
	NSCLC, NOS	4	4.88
	Others	3	3.66
Location of primary tumor	Upper or middle lobe	53	64.63
	Lower lobe	29	35.37
Location types	Central	53	64.64
	Peripheral	22	26.83
	Not specified	7	8.54
Interstitial pneumonia	Yes	7	8.54
	No	75	91.46
Clinical T classification	T1	5	6.10
	T2	20	24.39
	T3	24	29.27
	T4	33	40.24
Clinical N classification	N0	4	4.88
	N1	7	8.54
	N2	40	48.78
	N3	31	37.80
Clinical Stage	IIIA	26	31.71
	IIIB	36	43.90
	IIIC	20	24.39
Smoking history	Current or previous	38	46.34
	No	44	53.66
Hypertension	Yes	21	25.61
	No	61	74.39
Diabetes mellitus	Yes	13	15.85
	No	69	84.15
Treatment status	Initial treatment	29	35.37
	Recurrence or residual cancer	53	64.63
Total dose (Gy RBE)	Median (range)	72(48~78)	−
	48~72	48	58.54
	73.6~78	34	41.46
Combined Therapy	Immunotherapy	32	39.02
	Chemotherapy	47	57.32
	Targeted Therapy	8	9.76
Follow-up period(months)	Median (range)	31.5(2~66)	−

NSCLC, non-small cell lung carcinoma; KPS, Karnofsky Performance Status; Gy RBE, gray relative biological effectiveness.

Treatment history and maintenance therapy:Before CIRT, 29 patients (35.37%) received CIRT as their initial treatment, while 53 patients (64.63%) had a history of prior treatments, which included chemotherapy (38 cases, 46.34%), immunotherapy (28 cases, 34.15%), targeted therapy (8 cases, 9.76%), lobectomy (6 cases, 7.32%), radiotherapy (5 cases, 6.10%), and interventional therapy (2 cases, 2.44%). Following CIRT, 51 patients (62.2%) underwent maintenance therapy, which consisted of chemotherapy in 28 cases (TC in 4, TP in 6, PP in 10, others in 8), targeted therapy in 7 cases (osimertinib in 5, afatinib in 1, gefitinib in 1), and immunotherapy in 24 cases (tislelizumab in 10, sintilimab in 6, pembrolizumab in 7, bevacizumab in 1). It should be noted that a single patient may have received multiple types of prior treatments or maintenance therapies; therefore, the sum of individual treatment categories may exceed the total number of patients.

Carbon-ion radiotherapy regimens: The median CIRT dose was 72 Gy (range: 48–78 Gy), with the most frequent doses being 76 Gy (31 cases, 37.8%) and 72 Gy (30 cases, 36.5%). In the 72 Gy group, fractionations were 16 fx in 14, 15 fx in 5, 18 fx in 4, others in 7. In the 76 Gy group, fractionations were 19 fx in 10, 16 fx in 5, 17 fx in 5, 12 fx in 3, others in 8. The treatment patterns were as follows: CIRT alone in 18 cases (21.95%); CIRT combined with chemotherapy in 29 cases (35.37%); CIRT combined with targeted therapy in 2 cases (2.44%); CIRT combined with immunotherapy in 10 cases (12.20%); CIRT combined with chemotherapy and targeted therapy in 1 case (1.22%); CIRT combined with immunotherapy and targeted therapy in 5 cases (6.10%); and CIRT combined with immunotherapy and chemotherapy in 17 cases (20.73%). Detailed information is provided in the (**Supplementary Table 1**).

### 3.2 Treatment efficacy

**Figure 1:**
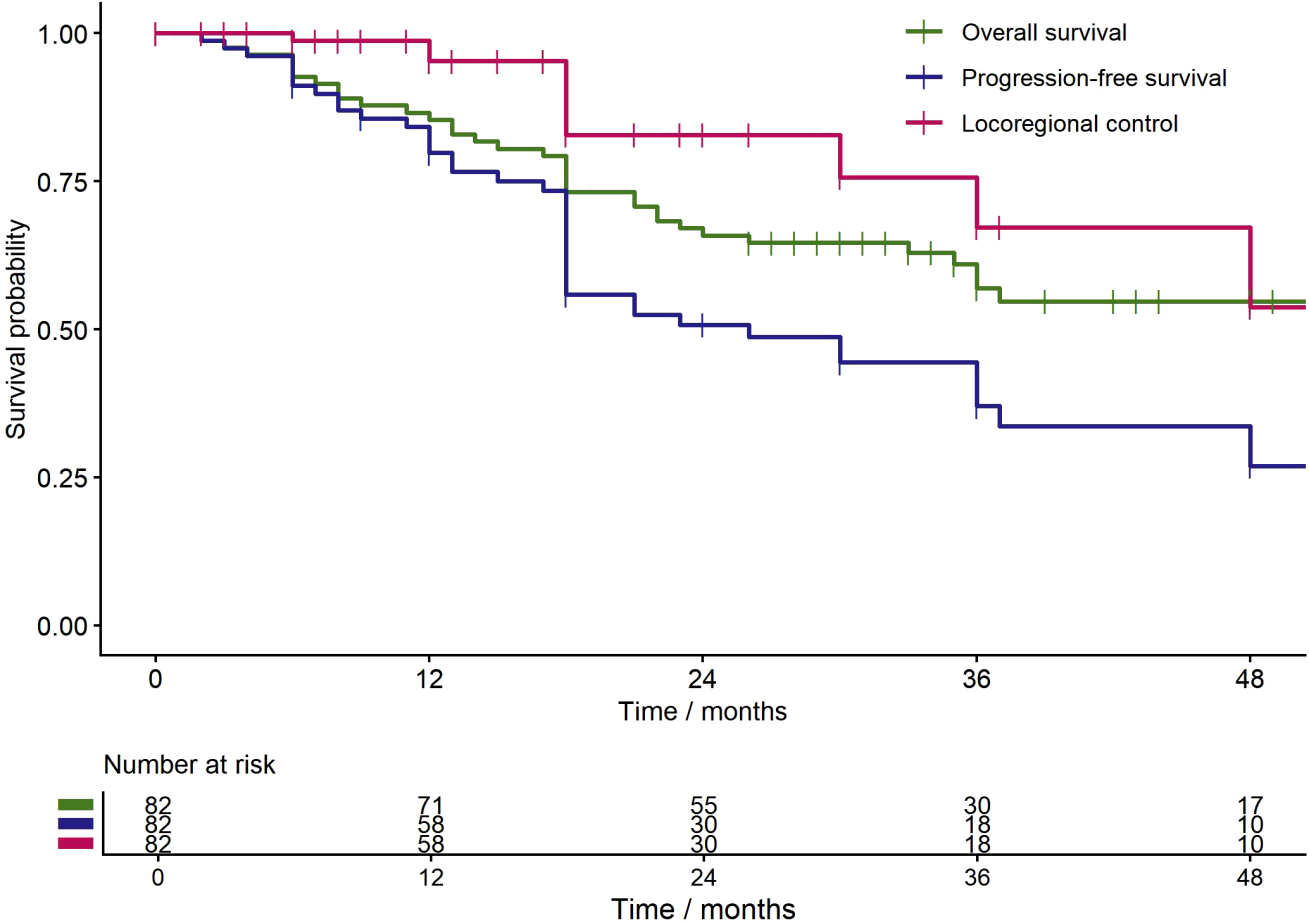
Kaplan-Meier curves of overall survival (OS), progression-free survival (PFS) and locoregional control (LRC) following carbon‐ion radiotherapy (CIRT).

At the conclusion of the follow-up period, 32 patients (39.0%) had succumbed, while 50 patients (60.9%) remained alive. The OS rates at 1 year and 2 years were 85.40% (95% CI: 78%-93.4%) and 65.90% (95% CI: 56.3%-77%), respectively. The PFS rates at 1 year and 2 years were 79.80% (95% CI: 71.1%-89.5%) and 50.80% (95% CI: 39.9%-64.6%), respectively. Additionally, the LRC rates at 1 year and 2 years were 95.30% (95% CI: 90.2%-100%) and 82.90% (95% CI: 73.1%-93.9%), as illustrated in **Figure 1**. The median PFS was 26 months, while the median OS has not yet been reached.

### 3.3 Adverse events

Treatment-related adverse reactions are summarized in **Table 2**. Five cases (6.1%) of grade 1 radiation pneumonitis were reported; 36 cases (43.9%) of grade 1 radiation esophagitis and 3 cases (3.7%) of grade 2 radiation esophagitis were reported; grade 1 radiation dermatitis in 2 cases (2.4%), grade 2 in 1 case (1.2%), and grade 3 in 1 case (1.2%). Adverse reactions of grade 3 or higher were primarily hematologic in nature, including grade 3 leukopenia in 7 cases (8.54%), grade 3 anemia in 5 cases (6.10%), grade 4 in 1 case (1.22%); grade 4 thrombocytopenia in 1 case (1.22%), grade 4 neutropenia in 4 cases (4.88%); grade 3 lymphopenia in 29 cases (35.37%), and grade 4 in 1 case (1.22%).

**Table 2:** Toxicities recorded during carbon‐ion radiotherapy (CIRT).

Grade	1 (%)	2 (%)	3 (%)	4 (%)	Total (%)
Pneumonitis	5(6.10)	0	0	0	5(6.10)
Esophagitis	36(43.90)	3(3.66)	0	0	39(47.56)
Dermatitis	2(2.44)	1(1.22)	1(1.22)	0	4(4.88)
Leucopenia	14(17.07)	9(10.98)	7(8.54)	0	30(36.59)
Anemia	8(9.76)	20(24.39)	5(6.10)	1(1.22)	34(41.46)
Thrombocytopenia	7(8.54)	6(7.32)	0	1(1.22)	14(17.07)
Neutropenia	10(12.20)	0	0	4(4.88)	14(17.07)
Lymphopenia	10 (12.20)	24(29.27)	29(35.37)	1(1.22)	64(78.05)

Regarding long-term adverse events, data were missing for 9 patients (11.0%). Among the remaining 79 patients, there were 3 cases (3.80%) of grade 1 radiation pneumonitis, 2 cases (2.53%) of grade 1 esophagitis, 2 cases (2.53%) of grade 1 dermatitis, and 1 case (1.27%) of grade 1 atelectasis.

### 3.4 Prognostic factors

**Figure 2:**
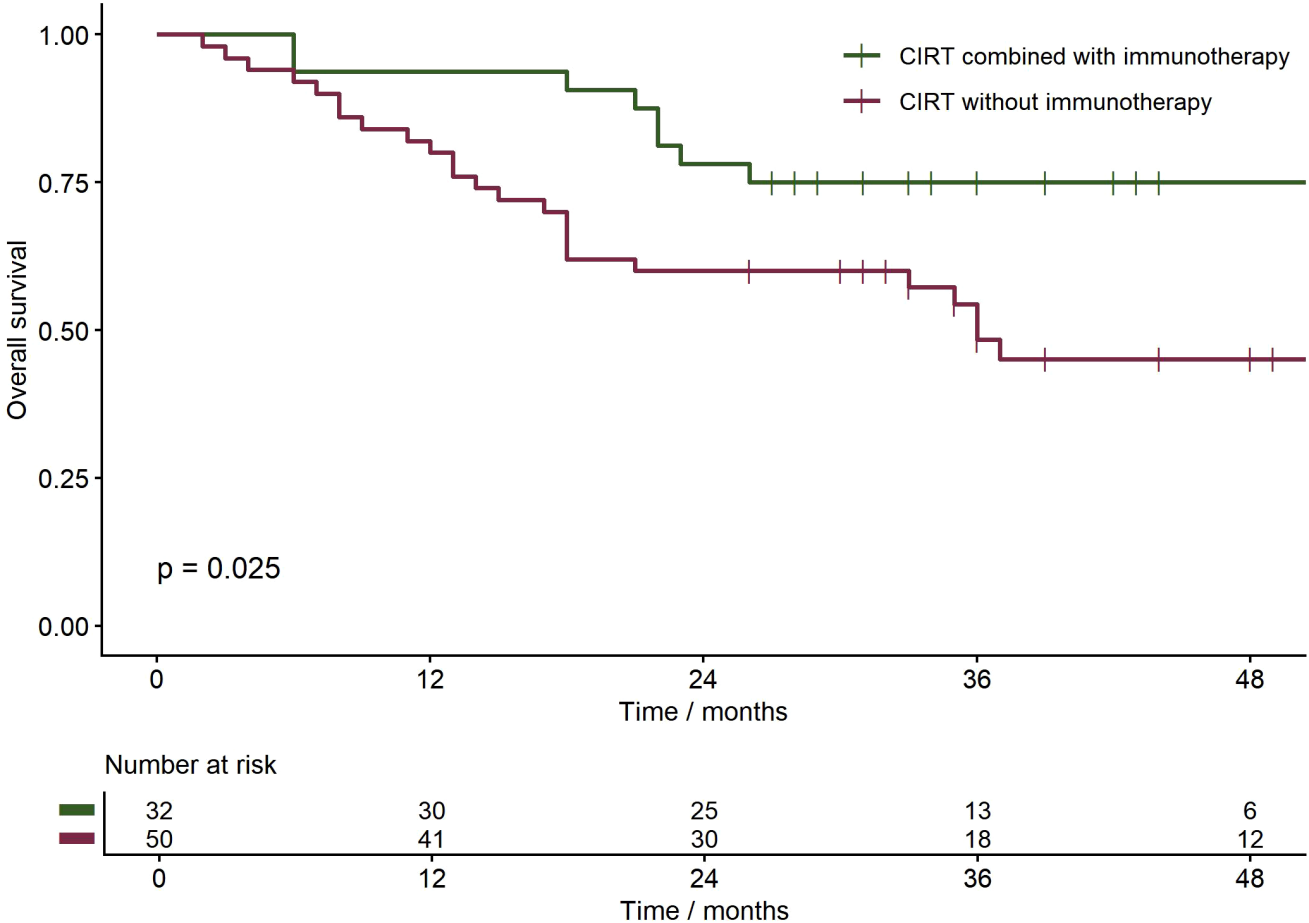
Kaplan-Meier curves of overall survival (OS) following carbon‐ion radiotherapy(CIRT) with and without immunotherapy.

Univariate and multivariate analyses were conducted to identify potential prognostic factors for OS, PFS, and LRC. The results of the univariate analysis showed that combined immunotherapy was a significant prognostic factor for OS (*P* = 0.03); smoking history and radiotherapy history were significant prognostic factors for PFS (*P* values of 0.092 and 0.069); and a history of immunotherapy was a significant prognostic factor for LRC (*P* = 0.078) (**Table 3**). In the multivariate analysis, CIRT combined immunotherapy remained an independent factor associated with improved OS (*P* = 0.025) (**Table 4**; **Figure 2**).

**Table 3:** Univariate analysis of overall survival (OS), progression-free survival (PFS) and locoregional control (LRC) rates.

Variable	OS		PFS		LRC	
	HR(95%CI)	P	HR(95%CI)	P	HR(95%CI)	P
Sex (male vs. female)	0.49 (0.15-1.61)	0.241	0.82 (0.34-1.95)	0.652	2.26 (0.76-6.67)	0.14
Age (<65 vs. ≥65 y)	1.15 (0.58-2.28)	0.69	1.43 (0.78-2.64)	0.25	1.99 (0.7-5.65)	0.197
Smoking History (no vs. yes)	1.69 (0.84-3.37)	0.138	1.7 (0.92-3.14)	0.09	0.8 (0.28-2.25)	0.67
AJCC stage (IIIA-B vs. IIIC)	0.72 (0.29-1.74)	0.459	1.03 (0.5-2.09)	0.942	1.22 (0.39-3.85)	0.732
Histology (others vs.adenocarcinom)	0.7 (0.33-1.52)	0.371	0.71 (0.36-1.4)	0.321	0.65 (0.19-2.16)	0.48
KPS score (<90 vs. ≥90Gy)	0.73 (0.37-1.44)	0.361	0.86 (0.47-1.58)	0.628	1.21 (0.43-3.42)	0.714
Chemotherapy History (no vs. yes)	0.85 (0.42-1.69)	0.639	0.87 (0.47-1.61)	0.657	1.33 (0.48-3.69)	0.577
Targeted Therapy History (no vs. yes)	0.88 (0.27-2.89)	0.834	0.67 (0.21-2.18)	0.51	0.71 (0.09-5.51)	0.747
Immunotherapy History (no vs. yes)	0.73 (0.34-1.57)	0.418	1.05 (0.55-1.99)	0.891	2.5 (0.9-6.9)	0.078
Lung Cancer Surgery History (no vs. yes)	0.31 (0.04-2.3)	0.255	0.51 (0.12-2.1)	0.35	0.72 (0.09-5.48)	0.75
Radiotherapy History (no vs. yes)	2.12 (0.64-7.02)	0.221	2.66 (0.93-7.57)	0.067	2.28 (0.28-18.21)	0.438
CIRT dose (<72 vs. ≥72Gy)	0.71 (0.34-1.46)	0.348	0.95 (0.51-1.77)	0.863	2.06 (0.73-5.79)	0.173
Combined Chemotherapy (no vs. yes)	0.71 (0.36-1.4)	0.318	0.7 (0.38-1.28)	0.242	1.15 (0.39-3.39)	0.801
Combined Targeted Therapy (no vs. yes)	0.24 (0.03-1.76)	0.16	0.38 (0.09-1.57)	0.18	0.53 (0.07-4.08)	0.545
Combined Immunotherapy (no vs. yes)	0.41 (0.19-0.92)	0.03	0.58 (0.3-1.12)	0.107	1.14 (0.41-3.16)	0.795

HR, hazard ratio; CI, confidence interval; OS, overall survival; PFS, progression-free survival; LRC, locoregional control; KPS, Karnofsky Performance Status.

**Table 4:** Multivariate analysis of overall survival (OS), progression-free survival (PFS) and locoregional control (LRC) rates.

Variable	OS		PFS		LRC	
	HR(95%CI)	P	HR(95%CI)	P	HR(95%CI)	P
Smoking History (no vs. yes)	−	−	1.69 (0.92-3.13)	0.092	−	−
Immunotherapy History (no vs. yes)	−	−	−	−	2.5 (0.9-6.9)	0.078
Radiotherapy History (no vs. yes)	−	−	2.64 (0.93-7.53)	0.069	−	−
Combined Immunotherapy (no vs. yes)	0.41 (0.19-0.92)	0.025	−	−	−	−

HR, hazard ratio; CI, confidence interval; OS, overall survival; PFS; LRC, locoregional control.

### 3.5 subgroup analyses

A total of 82 patients were enrolled in this study, including 18 patients in the CIRT monotherapy group and 64 patients in the combined treatment group. The two groups were balanced and comparable in most baseline characteristics, including age (*P* = 0.06), sex (*P* = 0.06), KPS score (*P* = 0.28), pathological diagnosis, tumor location, and clinical stage. However, the proportion of patients with a history of smoking was significantly higher in the combined treatment group than in the monotherapy group (60.9% vs. 27.8%, *P* = 0.02). Although the median total radiotherapy dose was 72 Gy in both groups, the distribution of doses differed significantly between the two groups (*P* = 0.01). Furthermore, preliminary data showed that the median OS was significantly longer in the combined treatment group than in the monotherapy group (33.0 months vs. 16.0 months, *P* = 0.01) (**Supplementary Table 2**). The 1-year and 2-year OS rates were 66.7% (95% CI: 48.1%–92.4%) and 38.9% (95% CI: 21.8%–69.4%) in the CIRT monotherapy group, and 90.6% (95% CI: 83.8%–98.1%) and 75.0% (95% CI: 65.1%–86.4%) in the combined treatment group, respectively (**Supplementary Figure 1**).

After adjusting for clinical stage, total dose, and smoking history, multivariate Firth’s Cox regression analysis showed that combined treatment was an independent protective factor for OS. Compared with the monotherapy group, the combined treatment group had a significantly reduced risk of death by 72% (HR = 0.28, 95% CI: 0.13%–0.61%, *P* = 0.002). Clinical stage and smoking history had no significant impact on OS in the multivariate model (**Supplementary Table 3**).

Interaction testing revealed no significant interactions between any prespecified subgroup variables and treatment allocation (*P* > 0.05), indicating that the OS benefit of combined treatment was highly consistent across different patient subgroups. Specifically, significant survival benefits of combined treatment were observed in elderly patients (≥65 years, HR = 0.28, *P* = 0.01), male patients (HR = 0.35, *P* = 0.01), and both high-dose and low-dose groups. Of note, the benefit of combined treatment was significant in patients with clinical stage A and stage C (both *P* < 0.05). However, in patients with clinical stage B, the beneficial trend did not reach statistical significance (HR = 0.57, *P* = 0.34), likely due to limited sample size or number of events, although the direction of benefit was consistent with the overall findings (**Supplementary Table 4**).

## 4 Discussion

To our knowledge, this study is the first to report on the treatment of stage III NSCLC using a domestically developed CIRT system in China, involving a substantial patient cohort. It provides a comprehensive assessment of key prognostic factors. In this study, the 1-year OS, PFS, and LRC rates for the 82 patients were 85.4%, 79.8%, and 95.3%, respectively, while the 2-year OS, PFS, and LRC rates were 65.9%, 50.8%, and 82.9%. The median PFS was 26 months, comparable to the findings of other recent CIRT studies (17, 18). A meta-analysis (19) indicated that for stage II-III NSCLC patients receiving particle therapy (including CIRT and PBT), the 2-year OS, PFS, and LRC rates were 61.3%, 37.9%, and 82.2%. Ma et al. (16) conducted a retrospective study involving 181 patients with stage III NSCLC treated with CIRT, with or without systemic therapy; the results demonstrated that the 1-year and 2-year OS, PFS, and LRC rates were 82.9% and 64.2%, 60.0% and 40.3%, and 79.7% and 66.1%, respectively. A Japanese study on carbon ion therapy alone for inoperable locally advanced NSCLC (20) reported 2-year and 3-year OS rates of 66.0% and 49.1%, with a median OS of 35.1 months.The 2-year and 3-year PFS rates were 37.5% and 28.3%, with a median PFS of 15.1 months, this indicates favorable survival outcomes and local control in the present study.

In this study, the overall acute adverse reactions were manageable (21, 22), with grade ≥3 adverse reactions predominantly comprising hematological issues, among which lymphopenia exhibited the highest incidence, accounting for 78.05%. This finding aligns with the results of Li et al. (23), which reported severe lymphopenia in patients with NSCLC undergoing proton-carbon ion radiotherapy. The study (23) demonstrated that, compared to photon intensity-modulated radiotherapy, proton-carbon ion radiotherapy significantly reduces the risk of severe lymphopenia by lowering the radiation dose to the thoracic spine and aorta, potentially serving as a reference for decreasing lymphopenia incidence following CIRT treatment. In this study, most patients received CIRT in conjunction with systemic therapy, suggesting that the high incidence of lymphopenia may also be attributed to this combination (24).

Furthermore, it is noteworthy that the incidence of esophagitis was relatively high, reaching 47.56%, with grade 1 esophagitis being the most prevalent, accounting for 43.90%. This indicates that prioritizing the prevention of esophagitis before and during treatment is essential. A study by Monti et al. (25) on dose patterns and predictive factors for radiation-induced esophagitis in NSCLC revealed that the upper and middle esophagus represent critical risk areas for the development of grade ≥2 radiation-induced esophagitis, identifying the average dose to the upper and middle esophagus (UME-Dmean) as a robust predictive indicator. Based on these findings, it is recommended that the upper and middle esophagus be treated as an independent organ at risk in CIRT planning, with dose constraints aimed at controlling UME-Dmean. This strategy seeks to minimize the risk of radiation-induced esophagitis while enhancing treatment tolerance and ensuring adequate target dose delivery. All adverse reactions reported were acute; data on long-term adverse reactions were limited and were not included in the analysis.

Multivariate analysis revealed that CIRT combined with immunotherapy was an independent favorable factor associated with improved OS. This is consistent with the findings of the PACIFIC study (26), where the 12-month PFS rate was 55.9% in the combination group versus 35.3% in the non-combination group, and the 18-month PFS rate was 44.2% versus 27.0%. Additionally, the study by Ma et al. (16) indicated that the median PFS for patients receiving immunotherapy after CIRT was 25.4 months, with 2-year OS and PFS rates of 73.1% and 54.2%, respectively, demonstrating that immunotherapy after CIRT significantly improves OS and PFS. In this study, a total of 32 patients (39.02%) received CIRT combined with immunotherapy, resulting in 1-year and 2-year OS rates of 93.8% (85.7%-100%) and 78.1% (65%-93.8%), respectively; the median OS has not yet been reached. Meng et al. (27) pointed out that CIRT has the potential to stimulate the immune activation of lymphocytes, such as T cells, NK cells, and dendritic cells (DCs); increase lymphocyte proliferation; enhance lymphocyte function; limit the induction of immunosuppressive cells such as myeloid-derived suppressor cells (MDSCs); and reduce the expression of immunosuppressive cytokines. Wang et al. (28) found that CIRT can induce the expression of the Klrk1 gene and activate the NKG2D/NKG2D-Ls pathway, thereby improving the infiltration and functional status of NK cells. Preclinical studies (29–31) have also demonstrated the efficacy and superiority of CIRT in combination with immunotherapy; however, clinical studies are relatively limited. Based on this, our study preliminarily suggests that CIRT combined with immunotherapy may be considered when the patient’s condition allows; however, its clinical benefits still require further validation through prospective studies.

This study also has several limitations: First, there were differences in the treatment regimens among the enrolled patients, as a unified CIRT fractionation scheme and systemic therapy regimen were not adopted. Second, there were missing follow-up data, making it impossible to thoroughly evaluate long-term adverse reactions and survival outcomes. Furthermore, since the median OS has not yet been reached, the relationship between adverse reactions and long-term prognosis remains unclear. Third, this study is a single-center retrospective analysis with a relatively limited sample size, which may introduce selection bias. Additionally, there is a lack of a prospective control group (such as a photon therapy group or a proton therapy group), making it impossible to directly demonstrate the clinical efficacy of carbon ion radiotherapy systems. Therefore, the generalizability of the study’s conclusions needs to be further validated through larger-scale, multi-center prospective studies.

In summary, CIRT demonstrated promising OS and manageable toxicity in patients with stage III NSCLC, with encouraging LRC. Patients receiving combined immunotherapy showed improved overall survival. Prospective studies are warranted to confirm this association and to define the optimal treatment strategy.

## Data Availability

The original contributions presented in the study are included in the article/**Supplementary Material**. Further inquiries can be directed to the corresponding authors.
