# Supplementary material for: Effectiveness and safety of carbon ion radiotherapy for stage III non-small cell lung cancer: a single-center retrospective study
Source: Front Oncol. 2026 Jun 30;16:1861846. doi: 10.3389/fonc.2026.1861846 (PMC13365176; doi:10.3389/fonc.2026.1861846)
Supplement: Supplementary file 1 [file DataSheet1.pdf]

Supplementary Information (SI)

Effectiveness and safety of carbon ion radiotherapy for stage III non-small cell lung cancer: a single-center retrospective study

Table.S1 Distribution of carbon-ion radiotherapy (CIRT) and its combination treatment regimens

Treatment Type	Regimen / Drug	Number	%
CIRT	-	18	21.95
CIRT + Chemotherapy	TP	11	13.41
	PP	5	6.1
	TC	4	4.88
	Nab-paclitaxel	3	3.66
	Nab-paclitaxel + Lobaplatin	3	3.66
	Nab-paclitaxel + Nedaplatin	1	1.22
	Nedaplatin + Etoposide	1	1.22
	Liposomal paclitaxel + Nedaplatin	1	1.22
CIRT + Targeted therapy	Anlotinib	1	1.22
	Osimertinib	1	1.22
CIRT + Immunotherapy	Cellular immunotherapy	5	6.1
	Pembrolizumab + Cellular immunotherapy	2	2.44
	Tislelizumab	2	2.44
	Pembrolizumab	1	1.22
CIRT + Chemotherapy + Targeted therapy	TP + Gefitinib	1	1.22
CIRT + Immunotherapy + Targeted therapy	Cellular immunotherapy + Osimertinib/Afatinib	5	6.1
CIRT + Immunotherapy + Chemotherapy	Tislelizumab + TP/TC/PP/Nab-paclitaxel	7	8.54
	Sintilimab+TP/Nab-paclitaxel+ Nedaplatin/Lobaplatin	6	7.32
	Cellular immunotherapy + TC/Pemetrexed	2	2.44
	Pembrolizumab + Cellular immunotherapy + PP	1	1.22
	Pembrolizumab + TC	1	1.22

CIRT, carbon-ion radiotherapy; TC, taxane + carboplatin; TP, taxane + cisplatin; PP, pemetrexed + platinum; Cellular immunotherapy includes CIK cells, NK cells, etc.

Table.S2 Baseline characteristics of patients with carbon-ion radiotherapy (CIRT) by treatment group (with vs. without systemic therapy)

**Characteristics**		**Monotherapy**	**Combined**	**p**
n		18	64	
Age (median [IQR])		68.50 [63.50, 71.50]	63.00 [56.00, 70.25]	0.063
Gender (%)	1	18 (100.0)	52 (81.2)	0.06
	2	0 (0.0)	12 (18.8)	
KPS_Score (median [IQR])		80.00 [80.00, 90.00]	90.00 [80.00, 90.00]	0.278
Pathological_Diagnosis (%)	1	10 (55.6)	34 (53.1)	0.291
	2	5 (27.8)	26 (40.6)	
	3	3 (16.7)	4 (6.2)	
Specific_Site (%)	1	12 (66.7)	41 (64.1)	1
	2	6 (33.3)	23 (35.9)	
T_stage (%)	1	2 (11.1)	3 (4.7)	0.142
	2	5 (27.8)	19 (29.7)	
	3	7 (38.9)	13 (20.3)	
	4	4 (22.2)	29 (45.3)	
N_stage (%)	0	1 (5.6)	3 (4.7)	0.936
	1	1 (5.6)	6 (9.4)	
	2	8 (44.4)	32 (50.0)	
	3	8 (44.4)	23 (35.9)	
Clinical_Stage (%)	A	6 (33.3)	20 (31.2)	0.742
	B	9 (50.0)	27 (42.2)	
	C	3 (16.7)	17 (26.6)	
Smoking_History (%)	0	5 (27.8)	39 (60.9)	0.017
	1	13 (72.2)	25 (39.1)	
Hypertension_History (%)	0	16 (88.9)	45 (70.3)	0.136
	1	2 (11.1)	19 (29.7)	
Diabetes_History (%)	0	16 (88.9)	53 (82.8)	0.723
	1	2 (11.1)	11 (17.2)	
Total_Dose (median [IQR])		72.00 [61.50, 72.00]	72.00 [72.00, 76.00]	0.008
Survival_Time (median [IQR])		16.00 [11.25, 31.75]	33.00 [25.25, 44.00]	0.011

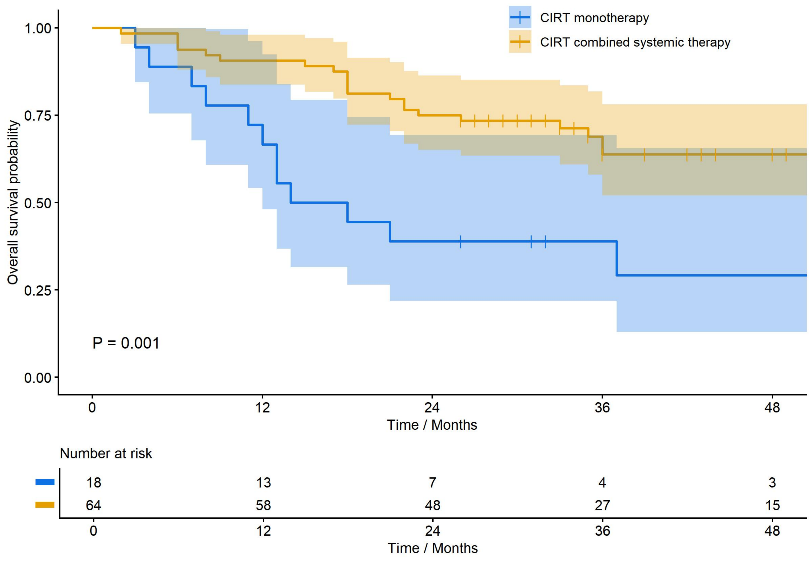

Figure. S1

Kaplan-Meier curves of overall survival (OS) following carbon-ion radiotherapy(CIRT) with and without systemic therapy.

Table.S3 Multivariate analysis of overall survival (OS) following carbon-ion radiotherapy (CIRT) with and without systemic therapy

Variable	HR_95_CI	P_value
Treatment_GroupCombined	0.28 (0.13-0.61)	0.002
Clinical_StageB	0.52 (0.24-1.12)	0.0936
Clinical_StageC	0.67 (0.24-1.66)	0.3961
Total_Dose	1.05 (1.00-1.12)	0.0673
Smoking_History1	1.56 (0.76-3.23)	0.2231

Table.S4 Subgroup analyses of overall survival (OS) following carbon-ion radiotherapy (CIRT) with and without systemic therapy

Subgroup	Level	N	P_value	P_interaction	HR_95_CI
Age_Group	>=65	41	0.009	0.585	0.28 (0.11-0.73)
Age_Group	<65	41	0.142		0.38 (0.13-1.46)
Gender	1	70	0.007		0.35 (0.17-0.74)
Gender	2	12	-		Not Evaluable
Clinical_Stage	A	26	0.028	0.056	0.28 (0.10-0.86)
Clinical_Stage	B	36	0.34		0.57 (0.19-1.94)
Clinical_Stage	C	20	0.029		0.17 (0.04-0.82)
Total_Dose_Group	High(>=72)	64	0.016	0.254	0.34 (0.16-0.81)
Total_Dose_Group	Low(<72)	18	0.024		0.14 (0.01-0.78)
